# Hepatoprotective activity of melittin on isoniazid- and rifampicin-induced liver injuries in male albino rats

**DOI:** 10.1186/s40360-021-00507-9

**Published:** 2021-07-03

**Authors:** Khalid Mohammed Naji, Bushra Yahya Al-Khatib, Nora Saif Al-Haj, Myrene R. D’souza

**Affiliations:** 1 Department of Chemistry, Faculty of Science, Sana’a University, Sana’a, Yemen; 2 Department of Chemical Ecology/Biological Chemistry, University of Konstanz, Universitätsstraße 10, 78464 Konstanz, Germany; 3 Department of Biology, Faculty of Science, Sana’a University, Sana’a, Yemen; 4 Department of Biochemistry, Mount Carmel College, Bengaluru, India

**Keywords:** Melittin, Bee venom, Hepatotoxicity, Tuberculosis, Rifampicin, Isoniazid

## Abstract

**Background:**

The present study investigated the ameliorative effect of melittin, a major polypeptide in the venom of honeybee (*Apis mellifera*), on isoniazid-(INH) and rifampicin-(RIF) induced hepatotoxicity in male albino rats.

**Method:**

Thirty rats (140-200 g) were divided into five groups (*n* = 6): normal control (NC) received normal saline orally (NaCl, 0.9%; toxic (T) group received INH + RIF (each rat received 100 mg/kg, p.o.); melittin (Mel15, Mel30) groups (each rat received 15 or 30 μg/kg s.c); and normal recovery (NR) group received INH + RIF (each rat received 100 mg/kg, p.o.). Blood and liver samples were collected for biochemical, hematological and histopathological studies respectively.

**Results:**

The administration of melittin was found to prevent the antitubercular drug-induced alterations in the diagnostic markers; reduced glutathione (GSH), direct bilirubin (DB), total bilirubin (TB), aspartate aminotransferase (AST), alanine aminotransferase (ALT), lactate dehydrogenase (LDH), alkaline phosphatase (ALP), and total serum protein (TSP). Besides, hematological alterations were significantly high in Mel groups when compared to the toxic group. The NR group exhibited lower levels of DB, TB, ALP, LDH and TSP. In addition, treatment with melittin offered protection in the NR group with respect to MDA levels.

**Conclusion:**

Evidence from this study suggests that melittin is beneficial for the prevention of acute hepatic failure in antitubercular drug-induced hepatoxicity and could be used as a potential therapeutic agent.

## Background

Tuberculosis is a highly communicable disease affecting over one-third of the world’s population and killing over 2 million people per year [1]. A meta-analytical study on the co-administration of isoniazid (INH) and rifampicin (RIF), the first-line drugs used for tuberculosis therapy indicated that they are associated with 2–6% hepatotoxicity [2], acute liver injury and high mortality rate [3]. These drugs mediate the generation of highly reactive oxygen species (ROS), which act as a trigger of lipid peroxidation and a means for destruction of the plasma membrane [4]. RIF actively induces CYP2E1, a member of the cytochrome P450 family responsible for the breakdown of environmental chemicals and carcinogens. It also increases INH induced toxicity by stimulating the formation of the toxic metabolite hydrazine via amidase pathway. Hydrazine then reacts with the sulfhydryl group of glutathione (GSH), depleting its levels within the hepatocytes and causing cell death [2]. Combination chemotherapy with INH-RIH was found to stimulate the conversion of INH to isonicotinic acid, another hepatotoxic product. The plasma half-life of acetyl hydrazine is further shortened by RIF as it is quickly converted to its active metabolites; thereby increasing the occurrence of liver necrosis [5].

Many traditional remedies employing herbal drugs continue to make a major contribution to health in terms of prevention and treatment of many diseases [6]. Venomous insects have long been used in scientific research and represent the basis of many traditional drugs [7]. Bee venom, a natural toxin produced by the honeybee (*Apis mellifera*) is known to relieve pain and treat inflammatory diseases, such as rheumatoid arthritis, in both humans and experimental animals [8, 9]. Bee venom comprises a variety of peptides, including melittin, adolpin, apamin, phospholipase A2 and mast cell degranulating peptide [10]. Melittin (MEL) is the principal toxin of bee venom accounting for approximately 50% of its dry matter. MEL is a small linear basic peptide with the chemical formula C_131_H_228_N_38_O_32_, twenty-six amino acid long and weighs 2847.5 Da [11]. It suppresses inflammation by inhibiting phospholipase (PLA) activity [12]. The protective effect of MEL on various inflammatory parameters has been reported by several investigators on acute liver inflammation [13], osteoarthritic chondrocytes [14], rheumatoid arthritis [15], and acute pancreatitis [16]. MEL was found to attenuate inflammation and fibrosis by inhibiting the NF-κB signalling pathway in liver fibrosis induced by thioacetamide. It decreases the rate of lethality, inhibits hepatocyte apoptosis and attenuates hepatic inflammatory responses. Recent reports in various disease models have proven the anti-inflammatory effects of MEL [17]. In the current study, we assessed the effects of melittin on anti-tuberculosis drugs-induced hepatotoxicity via biochemical analysis, haematological and histological examination.

## Methods

### Experimental animals

The study was carried out according to the guidelines prepared by the National Academy of Sciences and published by the National Institute of Health [18].

Thirty adults, male, healthy rats *Rattus rattus*, weighing 140–200 g were collected from the animal house, Biology Department, Faculty of Science, Sana’a University, Sana’a. The animals were housed in the research laboratory, Department of Pharmaceutics, University of Science and Technology, Sana’a for two weeks. Two animals were kept in each standard metallic cage (0.15 × 0.20 × 0.26 m) and maintained under room temperature (18–24 °C) with alternating 12 h light/dark cycle and 60% fresh air ventilation. The animals were provided with food pellets and water ad libitum. Collection trays were placed below the cages and daily cleaned. The amount of food and water taken by the rats were recorded every day, while the body weight was recorded every week.

### Experiment design and treatment

After the adaptation period, the rats were randomly divided into five groups of six animals:

i. ***Normal control (NC) group*:** Rats received normal saline orally (NaCl, 0.9%) daily, throughout the experimental period.
ii. ***Toxic (T) group*:** Each rat received INH (100 mg/kg body weight, p.o.), and RIF (100 mg/kg body weight, p.o.) for 21 days.
iii. ***Mel15 group*:** Each rat received INH (100 mg/kg body weight, p.o.), and RIF (100 mg/kg body weight, p.o.) for 21 days + MEL (15 μg/kg MEL, subcutaneously) for 15 days.
iv. ***Mel30 group*:** Each rat received INH (100 mg/kg body weight, p.o.), and RIF (100 mg/kg body weight, p.o.) for 21 days + MEL (30 μg/kg MEL, subcutaneously) for 15 days.
v. ***Normal recovery (NR) group***: Each rat received INH (100 mg/kg body weight, p.o.), and RIF (100 mg/kg body weight, p.o.) for 21 days. The animals were then allowed to recover for 15 days without any melittin.

All experiments were performed thrice within a period of 18 months during 2014–2016.

### Sample collection and preparation

#### Blood samples

After overnight fasting, all rats were anesthetized by slow subcutaneously administration of Ketamine/xylazine (50/5 mg/kg). At 08:00 AM, two sets of blood samples from the same animal were collected via the retro-orbital venous plexus using microhematocrit capillary tubes. The first set of blood sample was added to an empty tube to obtain serum for biochemical analysis. The second set of blood sample was added to an EDTA containing tube to obtain plasma for the hematological studies.

#### Tissue samples

All rats were sacrificed under anesthesia by cutting the neck using a sharp blade and then dissected. The liver was harvested, weighed and washed immediately with ice-cold saline to remove as much blood as possible. Each liver sample was divided into two parts - one part was used for preparing crude extracts, while the second part was fixed in 10% neutral-buffered formalin for histology analysis.

#### Preparing tissue extract

Briefly 10% extracts were prepared by homogenizing the tissue in ice-cold 0.1 M phosphate buffer (pH 7.4) with Teflon homogenizer at 3000 rpm for 10 min. The homogenate was centrifuged at 15,000 rpm for 30 min followed by lyophilization of the supernatant. The supernatant served as the source for reduced glutathione (GSH), lipid peroxidation (LPO) and total protein (TP) [19]. The preparation of tissues samples was carried out at the Tropical Disease Research Center–University of Science and Technology Hospital.

### Measurement of serum ALT, AST, ALP, TB and TSP

Serum biochemical parameters such as direct bilirubin (DB), total bilirubin (TB), aspartate aminotransferase (AST), alanine aminotransferase (ALT), lactate dehydrogenase (LDH), alkaline phosphatase (ALP), and total serum protein (TSP) were determined using kits supplied by Roche Diagnostics and the Roche/Hitachi Analyzer [20] at Al-Aulaqi Specialized Medical Laboratory, Sana’a.

### Biochemical analysis of tissues

The extent of lipid peroxidation was determined from the amount of thiobarbituric acid reactive substance (TBARS) formed by a reaction involving thiobarbituric acid [21]. The amount of reduced glutathione (GSH) [22] and total protein [23] were estimated in liver homogenate.

### Hematological assays

The red blood cells (RBCs), total white blood cells (T-WBCs) count, differential leukocytes count, platelets, hemoglobin (Hb), hematocrit (Hct), mean corpuscular hemoglobin (MCH), mean corpuscular hemoglobin concentration (MCHC), mean corpuscular volume (MCV), and red cell distribution width (RDW-CV) were measured [24] using automated Hematology System, Sysmex XT-2000i at Al-Aulaqi Specialized Medical Laboratory, Sana’a.

### Histological analysis

#### Preparation of tissue samples

The liver tissues were washed in ice normal saline solution, fixed in 10% formalin, dehydrated in graded alcohol (50–100%) and embedded in paraffin. The tissue was cut (3 μm) and stained with hematoxylin and eosin [25]. Sections were examined under a light microscope (Olympus microscope CX-21) and photographed by attached Olympus Camera DP21 (U-TV0.5XC-3).

#### Histopathological quantitative analysis

Quantitative analysis of the histopathological changes in the liver was averaged (*n* = 6 per group) using an ocular micrometer calibrated with a stage micrometer. The frequency of histopathological changes was based on an average obtained from an observation of 10-microscopic fields [26] with an area 625μm^2^ at 40x or 400x. The histology work was performed in the Histology Lab, at the University of Science and Technology.

### Statistical analysis

All data presented were expressed as mean ± standard deviation of three repeated set of the experiments. One-way analysis of variance (ANOVA) was used for analyzing statistical significances between the groups. This was followed by Tukey Multiple Comparisons using Prism 6 software (Graph Pad, San Diego, CA, USA). Values with *P* < 0.05 was considered significant.

## Results

### Body and liver weight

Result of body weight showed visible changes during the course of experiment. We recorded the body weight of each group by comparing the mean difference of final and baseline body weight. The NC group of animals had a mean body weight difference around 42.83 g. The T group showed a significant decrease 77.8% compared to the NC group, whereas, a significant increase of 75.18 and 88.25% was exhibited in Mel 15 and Mel 30 groups respectively compared to the T group (*P* < 0.05) (Table 1). At the end of the experiment, the mean of liver weight was 9.8 g for NC group. There were nonsignificant changes in the weight of the liver of the animals. The weight of liver increased by 36 and 48% in Mel 15 and Mel 30 groups respectively compared to the T group (*P* < 0.01) as shown in Table 1.

**Table 1:** Body weight and liver weight of male rats receiving melittin post isoniazid and rifampicin-induced hepatotoxicity

	NC	T	Mel15	Mel30	NR
Baseline weight (g)	151.8 ± 6.2	172.8 ± 14.1	176.8 ± 17.7	182.6 ± 16.5	172.87 ± 32.2
Final weight (g)	184.2 ± 18.3	174.7 ± 24.3	209 ± 20.5	220.5 ± 18.1	191.8 ± 41.6
Difference (g)	42.83 ± 5.78	9.5 ± 3.32	32.20 ± 8.46 ^b*^	37.8 ± 5.9 ^b*^	19.71 ± 8.22
Liver weight (g)	9.788 ± 1.05	7.57 ± 1.26	10.26 ± 0.99	11.28 ± 2.38 ^b**^	9.08 ± 2.23

Results are expressed as mean ± SD (n = 6)

*NC* control, *T* Toxic, Mel15: treated by 15 μg Melittin, *Mel30* treated by 30 μg Melittin, *NR* Normal recovery

^*^P < 0.05; ^**^P < 0.01; b: significant Vs T group

### Biochemical markers

A significant decrease in the levels of DB (85% for Mel15 and Mel30), TB (80% for Mel15 and 72% for Mel30), LDH (48% for Mel15 and 46% for Mel30) (*P* < 0.0001), ALT (47% for Mel15 and 41% for Mel30), ALP (43% for Mel15 and 42% for Mel30) (*P* < 0.001), AST (52% for Mel15 and 49% for Mel30), TSP (0% for Mel15 and 7.3% for Mel30) (P < 0.01) was seen compared to T group (Table 2). There was a significant decrease in DB, TB, ALP (*P* < 0.0001), ALT, AST (*P* < 0.05) and LDH (P < 0.001) levels in the Mel30 group when compared to the T group. On the other hand, there was a significant decrease in serum DB, TB (P < 0.0001), ALP, TSP (P < 0.001) and LDH (*P* < 0.05) in the NR group when compared with the T group (Table 2).

**Table 2:** Biochemical parameters of male rats receiving melittin post isoniazid and rifampicin-induced hepatotoxicity

	NC	T	Mel15	Mel30	NR
DB (μmol/l)	1.0 ± 0.451	7.05 ± 1.84^a****^	1.0 ± 0.38^b****^	1.0 ± 0.27 ^b****^	1.0 ± 0.31^b****^
TB (μmol/l)	1.6 ± 0.94	10.45 ± 2.13^a****^	2 ± 1.05^b****^	2.82 ± 1.49 ^b****^	2.87 ± 0.89 ^b****^
ALT (U/L)	26.83 ± 11.44	73.5 ± 16.99^a***^	38.5 ± 14.27^b**^	44.33 ± 15.93^b*^	50.33 ± 17.44
AST (U/L)	92.17 ± 31.64	288.3 ± 147.7^a**^	137.7 ± 15.81^b*^	147 ± 32.83^b*^	183.2 ± 79.05
ALP (U/L)	187.2 ± 25.86	286.5 ± 53.07^a***^	164.2 ± 19.59^b****^	166.5 ± 18.41^b****^	189 ± 27.41^b***^
LDH (U/L)	762 ± 63.68	3046 ± 768.1^a****^	1577 ± 338.4^b***^	1640 ± 693.3^b***^	2155 ± 261.2^b*^
TSP (g/L)	66 ± 8.37	75.5 ± 0.84^a**^	76 ± 1.67	70 ± 2.76	63.5 ± 2.51^b***^

Results are expressed as mean ± SD (n = 6)

*NC* control, *T* Toxic, *Mel15* Melittin15μg, *Mel30* Melittin30μg, *NR* Normal recovery

^*^P < 0.05; ^**^P < 0.01; ^***^P < 0.001; ^****^P < 0.0001

a: significant Vs NC group, b: significant Vs T group

### Antioxidant markers and total protein

**Fig. 1:**
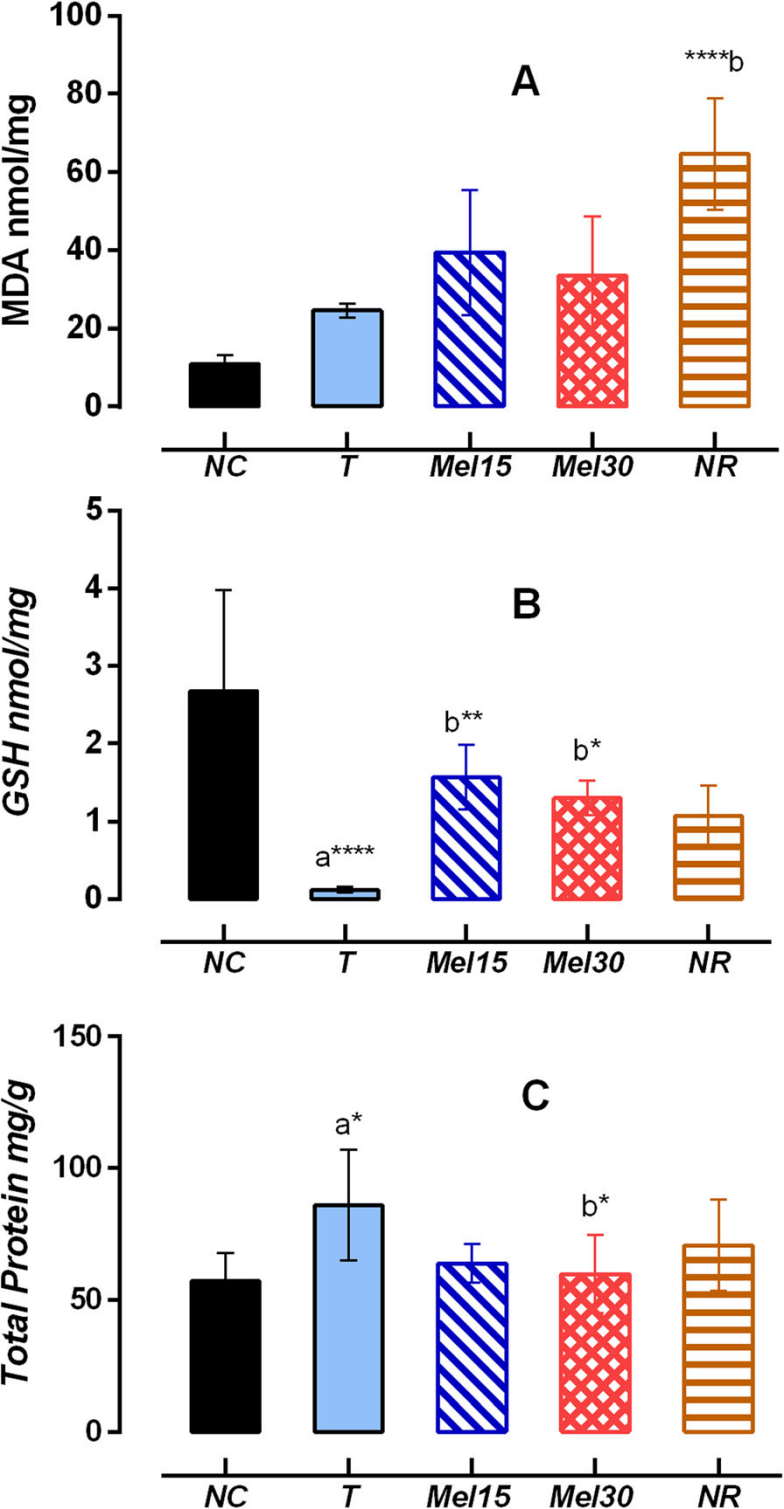
Effect of Melittin in the levels of MDA, GSH and TP in post isoniazid and rifampicin-induced hepatotoxicity. Results are expressed as mean ± SD for at least six rats per group. NC: control; T: Toxic; Mel15: Melittin15μg; Mel 30: Melittin30μg; NR: Normal recovery; *P < 0.05; **P < 0.01; ***P < 0.001; ****P < 0.0001. a: significant vs NC group, b: vs T group

A significant increase in MDA was demonstrated in NR (P < 0.0001) when compared with T group. On the other hand, there was an insignificant reduction in liver MDA in Mel15 and Mel30 groups (Fig. 1a). Furthermore, a significant increase in GSH level was observed in the Mel15 (*P* < 0.01) and Mel30 (*P* < 0.05) groups compared with the T group after 15 days of treatment. No significant difference in GSH level was observed in the NR group compared to the T group (Fig. 1b).

Also, a significant decrease in the total protein content was noted in Mel30 group (*P* < 0.05) when compared with T group. No significant changes between the rats that received Mel15 and T group was seen (Fig. 1c).

### Haematological parameters

After administration of MEL for 15 days, a significant decline in BAS and MCHC (*P* < 0.0001) when compared to T group was reported. A significant increase was found in Mel30 group for WBCs (P < 0.0001), NEU, Hb, PLT and RDW- CV (*P* < 0.01), Hct and MCV (*P* < 0.001), EOS and MCH (*P* < 0.05), while BAS and MCHC were significant decreased (P < 0.05) in comparison with T group. On the other hand, there was a significant increase in WBCs and Hb (P < 0.05), NEU (*P* < 0.001), EOS, Hct, MCH (*P* < 0.01) and MCV (P < 0.0001). There a significant decrease in BAS, MCHC (*P* < 0.0001) in the NR group compared to the T group (Table 3). Moreover, LYM and MON in groups NC, T, Mel15, Mel30 and NR had no statistical changes.

**Table 3:** Complete blood count in male rats that received melittin post isoniazid and rifampicin-induced hepatotoxicity

	NC	T	Mel 15	Mel 30	NR
WBCs(×10^3^/μl)	8.75 ± 1.19	5.45 ± 2.09	11.91 ± 1.32^b****^	14.13 ± 2.78^b****^	9.525 ± 2.00^b*^
LYM (%)	70.12 ± 7.82	79.23 ± 7.44	75.77 ± 4.19	70.52 ± 9.36	67.67 ± 11.65
MON (%)	4.55 ± 2.20	4.433 ± 2.12	7.117 ± 2.38	5.1 ± 2.18	5.75 ± 1.86
NEU (%)	19.5 ± 4.33	5.633 ± 1.09^a**^	13.73 ± 2.24	21.17 ± 8.98^b**^	22.85 ± 9.88^b***^
EOS (%)	1.15 ± 0.23	1.22 ± 0.26	3.23 ± 1.55^b*^	3.48 ± 1.43^b*^	3.65 ± 1.36^b**^
BAS (%)	0.08 ± 0.04	9.483 ± 4.41^a****^	0.15 ± 0.05^b****^	0.1 ± 0.0^b****^	0.08 ± 0.04^b****^
RBCs) × 10^6^/μl)	9.033 ± 0.852	7.755 ± 0.26 ^a**^	7.90 ± 0.22	7.952 ± 0.69	7.305 ± 0.34
Hb (g/dl)	15.18 ± 0.80	13.03 ± 0.89^a**^	14.88 ± 0.32^b**^	15.17 ± 0.74^b**^	14.5 ± 1.17^b*^
Hct (%)	48.78 ± 2.67	40.67 ± 2.28^a***^	48.22 ± 1.90^b**^	49.17 ± 2.47^b***^	47.6 ± 4.46^b**^
MCV (ƒL)	65.3 ± 3.31	52.4 ± 1.69^a****^	61.03 ± 2.11^b**^	62.12 ± 3.81^b***^	65.23 ± 5.48^b****^
MCH (pg)	20.32 ± 0.79	17.52 ± 0.65^a***^	18.85 ± 0.35	19.12 ± 1.15^b*^	19.85 ± 1.27^b**^
MCHC (g/dL)	31.13 ± 0.41	33.37 ± 0.38^a****^	30.90 ± 0.99^b****^	30.83 ± 0.36^b****^	30.5 ± 0.97^b****^
PLT (×10^3^/μl)	777 ± 68.9	427.5 ± 146.5^a**^	827.8 ± 76.3^b***^	794.5 ± 130.6^b**^	607.7 ± 246.4
RDW-CV (%)	18.22 ± 2.1	24.35 ± 2.1^a****^	27.1 ± 1.84	28.65 ± 1.93^b**^	24.75 ± 1.56

Results are expressed as mean ± SD (n = 6)

*WBCs* White Blood Cells; *Lym* lymphocytes; Mon: Monocytes; *Neu* Neutrophils; *EOS* Eosinophils; *Bas* Basophils; *RBCs* Red Blood Cells; *Hb* Hemoglobin; *Hct* Hematocrit; *MCV* Mean corpuscular volume; *MCH* Mean Corpuscular Hemoglobin; *MCHC* Mean corpuscular Hemoglobin concentration; *PLT* platelets; *RDW-CV* Red blood cell distribution width - Coefficient of Variation

*NC* control, *T* Toxic, *Mel15* Melittin15μg, *Mel 30* Melittin30μg, *NR* Normal recovery

^*^P < 0.05; ^**^P < 0.01; ^***^P < 0.001; ^****^P < 0.0001. a: significant Vs NC group, b: significant Vs T group

### Histopathology

**Fig. 2:**
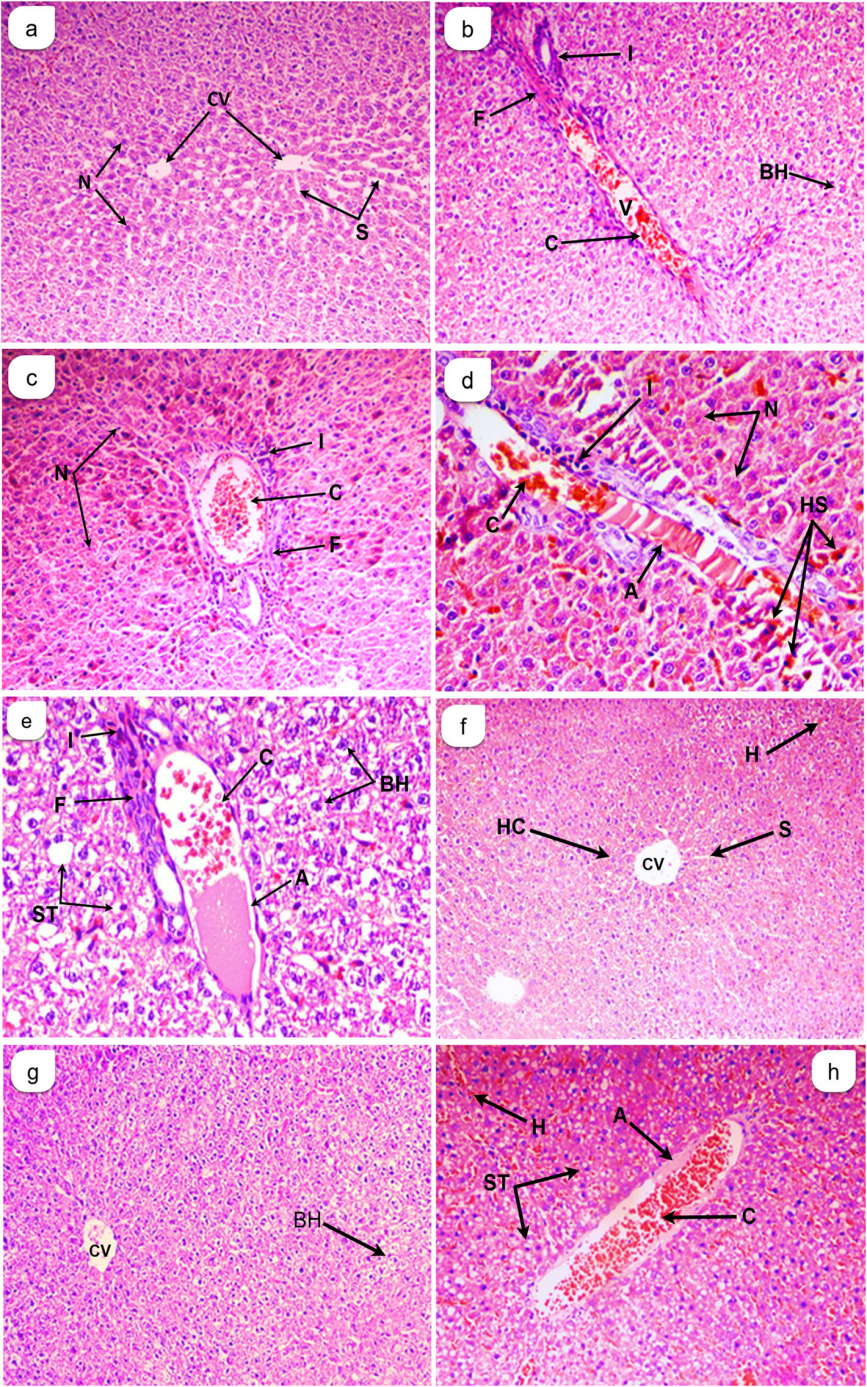
Cross sections in liver of rats showed the effect of Melittin on liver tissues in post isoniazid and rifampicin-induced hepatotoxicity. (**a**): Normal control (NC); (**b**): toxic group (T); (**c**): treated group 15 μg Melittin; (**d**): treated group 30 μg Melittin (Mel 30); (**e**): Normal recovery group (NR). (**a**): Normal group (N), 200x; (**b & c**): Toxic group (T), 200x; (**d & e**): Toxic group(T) 400x; (**f**): Mel 15 mg, 200x; (**g**): Mel 30 mg, 200x; (**h**): Normal recovery group (NR) 200x. *Histological abbreviations*: Amyloids (A), Congested blood vessel (C), Ballooned hepatocytes (BH), Inflammation (I), Necrosis (N), Vasodilation (V), Fibrosis (F), Hemorrhage (H), Steatosis (ST), Hepatocytes (HC), Central vein (CV), Sinusoid (S) and Hemosiderin-laden macrophages (HS)

A qualitative assay for histological lesions showed exhibited a central vein and normal hepatocytes (Fig. 2a). Liver sections of hepatotoxic group revealed severe histopathological lesions as shown in Table 4 and Fig. 2 (b, c, d & e). Mel15 group demonstrated significantly lower scores of hemorrhage and congestion (*P* < 0.001), ballooned hepatocytes and inflammation (*P* < 0.01), while the greatest decrease was detected for necrosis, fibrosis and amyloids (*P* < 0.0001) when compared to T group as shown in Table 4 and Fig. 2f.

**Table 4:** In situ evaluation of liver from rats that received melittin post isoniazid and rifampicin-induced hepatotoxicity

	NC	T	Mel15	Mel30	NR
Amyloids	–	++++	–	–	+
	1.33 ± 0.52	17.5 ± 6.47^a****^	2.67 ± 1.21 ^b****^	1.5 ± 0.55^b****^	3.83 ± 1.72 ^b****^
Congestion	+	++++	+	+	+++
	1.33 ± 0.52	6.67 ± 1.36 ^a****^	2.4 ± 1^b***^	2.17 ± 0.98^b***^	5.17 ± 2.56
Ballooned hepatocytes	–	++++	+	++	++
	4 ± 2	86 ± 39.43^a****^	30 ± 11.65^b**^	49.4 ± 17.81	54.3 ± 19.79^a**^
Inflammation	–	++++	++	++	++++
	0.83 ± 0.41	4.5 ± 0.84^a****^	2.5 ± 1.0^b**^	2.5 ± 1.2^b**^	4.4 ± 1.0
Necrosis	+	++++	+	+	+++
	13.17 ± 4.8	64.83 ± 17.2^a****^	23.17 ± 10.1^b****^	22.33 ± 4.1^b****^	43.5 ± 14.3^b*^
Vasodilation	–	++++	++	+++	++++
	1.8 ± 0.41	16.3 ± 4^a***^	11.2 ± 4.7	12.5 ± 4.76	20.3 ± 7.2
Fibrosis	–	+++	–	+	++++
	0.83 ± 0.41	6.83 ± 2.0^a****^	1.3 ± 0.5^b****^	1.8 ± 0.4^b***^	7.3 ± 3.2
Hemorrhage	–	+++	+	++	++++
	1.5 ± 0.55	5.5 ± 0.55^a****^	2.13 ± 0.95^b***^	4.5 ± 2.1	9.167 ± 1.17^b***^
Steatosis	–	+	–	–	++++
	1.3 ± 0.5	16.5 ± 5.1	3 ± 1.1	6.67 ± 1.6	48.5 ± 19.9^b****^

Results are expressed as mean ± SD (n = 6)

The histopathological changes were based on the average obtained from an observation of 10-microscopic fields with an area 625μm^2^ at 40x or 400x

*NC* control, *T* Toxic, *Mel15* Melittin15μg, *Mel 30* Melittin30μg, *NR* Normal recovery

Damages graded as follows: -, absent; +, trace (1–25%); ++, mild (25–50%); +++, moderate (50–75%); ++++, severe (75–100%). ^*^P < 0.05; ^**^P < 0.01; ^***^P < 0.001; ^****^P < 0.0001

a: significant Vs NC group, b: significant Vs T group

Furthermore, Mel30 group had significantly decreased scores for congestion and fibrosis (P < 0.001), and inflammation (P < 0.01). The greatest decrease was however detected with necrosis and amyloids (P < 0.0001) as compared to T group (Table 4 and Fig. 2g). In addition, the NR group had significantly decreased scores of hemorrhages (P < 0.001), and steatosis (*P* < 0.05), while showing significant reduction in necrosis (P < 0.05), and amyloids (P < 0.0001), when compared with T group (Table 4 and Fig. 2h).

## Discussion

The present investigation examines the protective effects of the peptide, melittin a major component of bee venom against hepatotoxicity induced by the co-administration of Isoniazid (INH) + Rifampicin (RIF) in adult male albino rats for 21 days. A dose-weight dependent increase indicated that Mel30 was more effective than Mel15in treatment. Thus, it could imply that treatment with MEL improves the food intake, food palatability, metabolic processes, and consequently increased the body weight and liver/body weight ratio of rats that received INH + RIF rats.

Increase in activity of the enzymes ALT and AST is indicative of massive hepatocellular disease and is linked to acute hepatic necrosis [27]. Chronic liver disease is diagnosed by a large increase of mitochondrial AST in the serum following extensive tissue necrosis [28]. The increase in ALP activity in the T group (Table 2) is related to improper bile flow through the gallbladder and bile duct into the intestine [29], leaky tight junctions between the canaliculi wall cells [30], overproduction and release of ALP into the blood due to hepatobiliary injury and cholestasis [31]. The decline reported in Mel15 and Mel30 samples indicates a positive effect of MEL on ALP in the treatment used which reflect the role of melittin on reduction of cholestasis and hepatobiliary cell injury through reduction in ductular reactions and cholangiocyte proliferation [32]. The high serum levels of LDH reported in the T group is an important index of liver cell damage probably due to hepatocellular necrosis [33]. Mel15 and Mel30 treatment demonstrated a considerable decline in serum LDH activity indicating its contribution towards the protection of liver epithelial cells from damage through the inhibition of inflammatory cytokines and apoptosis as reported previously [34]. It could also be suggested that the administration of MEL may prevent liver damage related to the maintenance of plasma membrane integrity, thereby suppressing the leakage of enzymes through membranes. Additionally, MEL may also exhibit hepatocurative activity against the hepatotoxicity induced by a combination of INH and RIF drugs [35]. Mel15 and Mel30 treatment reduced the elevation of AST and ALT activities; with MEL groups showing greater improvement than the NR group. From the results obtained, Mel15 treatment provided a better reduction in serum levels of the AST and ALT when compared to Mel30 treatment. This indicates that Mel at 15 μg/kg is more efficacious than 30 μg/kg for reducing the biochemical parameters induced by INH + RIF. This may correspond to the adverse effect of high amount of melittin that accumulates and leads to cell lysis [32]. Although Melittin treatment significantly reduced the liver enzymes levels, they remain relevantly high compared to control (NC), which may have resulted from the massive damage of liver generated by INH + RIF.

Anti-tubercular drugs through oxidative stress causes cellular damage as well as dysfunction of the hepatic antioxidant defense system [36]. Enhanced susceptibility of the hepatocyte cell membrane to anti-tubercular drugs induces peroxidative damage which increases the activity of diagnostic marker enzymes (Table 2) in the systemic circulation_._ Mel15 and Mel30 treatment exhibited a statistically significant (*P* < 0.0001) decline in MDA levels in NR group when compared to T group (Fig. 1) indicating its ability to prevent hepatocellular damage [37]. The increase in GSH (Fig. 1) upon Mel15 and Mel30 treatment compared to T group suggests its capability of preventing the depletion of antioxidant defenses by shifting the prooxidant-antioxidant balance to negate oxidative stress-induced cell death [38]. These findings were in similarity with results in GalN/ LPS- induced liver injury [39].

Anti-tubercular drugs have been recorded to cause hemolytic and aplastic anemia by the depression of the bone marrow [40, 41], with INH and its metabolite in particular affecting the erythroid precursor cells [42, 43]. Furthermore, INH binds to pyridoxine (vitamin B6), depleting its levels and causing anemia [44]. The increase in hematological parameters such as WBC, MON, NEU, EOS, Hct, PLT and RDW-CV in Mel15 and Mel30 groups when compared to the T group and NR group is suggestive of the role of MEL in ameliorating the hepatotoxicity induced by INH and RF [45]. Decrease in Hb levels is probably due to INH-mediated inhibition of heme synthesis by depressing δ-aminolevulinic acid (ALA) synthetase, the first and rate-limiting enzyme in the heme synthesis pathway [46]. The result of this study revealed that MEL treatment could reverse the decline in Hb levels seen in T and NR groups (Table 3). The decrease in MCV and MCH, indicative of microcytic hypochromic anemia is reported in all groups. Mel15 and Mel30 treatment could reverse this condition even though NR exhibited a higher recovery. However, Mel15 and Mel30 treatment did reduce MCHC when compared to T and NR group (Table 3). The significant decrease in platelets reported in T and NR groups (Table 3) is related to increased thrombocytopenia [46] possibly by RIF binding non-covalently to thrombocyte membrane glycol-proteins IIb/IIIa or Ib/IX to produce compound epitopes or induce conformational changes [47]. Treatment with Mel15 and Mel30 was shown to increase PLT (Table 3) providing better chances of reversing thrombocytopenia-induced by INH and RH. Basophil count significantly raising is very rare, but if seen, indicates myeloproliferative disorder or other more obscure causes [48]. Treatment with Mel15 and Mel30 reversed the increase in BAS, reducing it to almost NC levels (Table 3) which refers the high power of melittin as an antiinflammation agent. The increase in WBCs, NEU, EOS, Hb, Hct, MCV, MCH and PLT suggests a positive effect of MEL against blood coagulation, triggering of erythrocytes formation and the regeneration of leucocytes and erythrocytes [49]. The increase of EOS in Mel15 and Mel30 groups may be due to the allergenic nature of melittin. This result was affirmed when several patients taking MEL complained from allergy symptoms [50].

A considerable decline was reported with Mel15 and Mel30 treatments This explains the decrease in TP levels in the tissues of rats that received MEL (Fig. 1c). Vasodilation observed to be the highest in T group (Table 4), induced by the action of histamine on vascular smooth muscle leads to increased permeability of the microvasculature, causing the outpouring of protein-rich fluid into extravascular tissues [19]. The vessels diameter decreased upon Mel15 and Mel30 treatment resulting in decreased concentration of red blood cells in small vessels, decreased viscosity of blood and reduced chances of engorgement of the small vessels. Thereby, the condition of stasis observed in the T group is reduced in MEL groups. Ballooned cells seen in large number in the T group are formed by excess water accumulation in the vacuoles as a result of the failure of active membrane transport due to loss of ATP as the energy source [51]. Number of ballooned cells in the liver of the Mel15 and Mel30 groups reduced when compared to T and NR groups (Table 4), suggesting that melittin possesses ameliorating effect of the active membrane transport.

In our study, necrosis observed as one of the most distinct pathological features of INH + RIF in liver sections. It was seen to decline upon Mel15 and Mel30 treatments when compared to T and NR groups, thereby reflecting the protective role of melittin from the necrosis produced by INH-RIF (Table 4). Fibrosis was reported to be highest in NR and T groups (Table 4, Fig. 2) possibly due to the activation of hepatic stellate cell (HSC) and their phenotypic transformation into myofibroblasts, possessing pro-fibrogenic role [52, 53]. The condition of fibrosis was found to decline considerably in groups that received Mel15 and Mel30 (Table 4) which indicates the role of melittin in preventing INH-RIF- induced liver fibrosis by inhibiting liver inflammation. The behavior was also reported in previous experiments that showed significant inhibitory action in melittin groups when compared to the toxic groups [54, 55]. Melittin was found to suppress proinflammatory cytokine expression via nuclear factor (NF)-κB signaling pathway in hepatic stellate cells cultured with TNF-α [54]. Furthermore, the fibrotic gene expression in response to thioacetamide- induced liver fibrosis was found to decrease in the presence of melittin [54]. The anti-apoptotic nature of melittin was illustrated in TNF-α/actinomycin D-induced apoptosis in hepatocytes where it prevented TNF-α/Act D-induced activation of bcl-2 family of proteins, caspase and poly ADP-ribose polymerase (PARP)-1 [55]. In addition, it was reported that the NF-κB decreased on account of degradation of phosphorylation of IκB kinase (p-IKK) as well as NF-κB DNA binding activity in TNF-α/Act D-treated hepatocytes. The suppression in apoptosis could be explained by treatment with melittin [55].

Melittin also protects against LPS-induced acute kidney injury by attenuating the production of renal and systemic levels of cytokines, preventing immune cell accumulation and by suppression of NF-κB pathway. It decreases the expression of NADP oxidase-4 while enhancing nuclear factor erythroid-2 related factor (Nrf2) mediated antioxidant defenses. This change inhibits apoptotic and necrotic renal cell death following treatment with LPS [56].

The decrease in amyloid deposition in hepatocytes in rats that received Mel can be explained by the work done on LPS-induced inflammation in BV2 microglial cells [57]. In this study, hepatocytes co-treated with LPS and melittin exhibited decreased expression of IL-6, IL-1β, and TNF-α and a corresponding improvement in motor function compared to the control [58]. This behavior was explained by improved proteasomal function along with decline in TNF-α and the corresponding decrease in Map 2, a marker for neuronal cell function [58].

Our results further strengthened the role of melittin in reducing the high rate of lethality, chronic liver injury, attenuating hepatic inflammatory responses and inhibiting hepatocyte apoptosis. Thus, it is clear from our findings that melittin might possess an ameliorative role in biological, biochemical, hematological and histological alterations against hepatotoxicity, induced by INH and RF. Also, Melittin can prevent necrosis and fibrosis in many diseases. These results present building blocks for performing further studies that will help in the treatment of some degenerative disease such as Alzheimer disease.

## Conclusions

Novel drugs often developed from natural products such as toxins from bees, snakes and scorpions provide therapeutics for the treatment of inflammatory and neurodegenerative diseases. It is evident from this study that melittin protects isoniazid and rifampicin induced hepatotoxicity. Its use as an anti-inflammatory agent will depend on the development of innovative research protocols to validate its efficacy and safety. Modification of the toxic peptide could overcome the associated ill-effects and pave a way for promoting novel pharmaceutical agents. We propose that melittin could be used as a potential therapeutic agent for attenuating acute liver injury and hepatic failure provided prerequisites are met to evade adverse effects.

## Data Availability

The datasets supporting the conclusions of this article are included within the article.

## Abbreviations

INH: Isoniazid
RIF: Rifampicin
MEL: Melittin
DTNB: 1,2 dithio-bis nitrobenzoic acid
TBA: Thiobarbituric acid
ALT: Alanine aminotransferase
AST: Aspartate aminotransferase
ALP: Alkaline phosphatase
LDH: lactate dehydrogenase
TSP: Total serum protein
MDA: Malondialdehyde
GSH: Reduced glutathione
